# Chronic Granulomatous Reaction to Semi-permanent Eyebrow Tint

**DOI:** 10.7759/cureus.44070

**Published:** 2023-08-24

**Authors:** Saba Suleman, Maria Villegas, Thomas Davis, Charles S Stevens, Patricia Castaneda

**Affiliations:** 1 Internal Medicine, Baylor Scott & White All Saints, Fort Worth, USA; 2 Dermatology, University of Texas Rio Grande Valley School of Medicine, Harlingen, USA; 3 Dermatology, Sagis Diagnostics, Dallas, USA; 4 Dermatology, South Texas Dermatopathology, San Antonio, USA

**Keywords:** medical and cosmetic dermatology, dermatology, granulomatous reaction, granulomatous, eyebrow tint, semi-permanent cosmetics, micropigmentation, microblading

## Abstract

Eyebrow micropigmentation, also known as eyebrow microblading or embroidery, is a new technique in the field of semi-permanent cosmetics that are used for therapeutic and aesthetic purposes to recreate eyebrow structure and definition. It uses synthetic pigment that is deposited through fine needles into the papillary dermis and remains till the body metabolizes the pigment and clinically fades away by 12-18 months. Similar to other tattooing procedures, microblading involves risks including local inflammation, infection, allergic contact dermatitis, and granulomatous reactions that can occur from months to years after the procedure. We describe herein a case of a 49-year-old female who has persistent erythematous and indurated plaques on both eyebrows after a microblading procedure performed over a year and a half prior to her initial visit.

## Introduction

Eyebrows are one of the most prominent facial features for an individual due to the many functions they serve. Their architecture helps shape the face, accentuate the eyes, and express powerful nonverbal communication. While people cannot change the shape of their faces, they can enhance and reshape their eyebrows to alter their appearance. A technique known as microblading also referred to as “eyebrow embroidery,” originated in Asia and is continuing to gain popularity across the globe [1]. A key motivator to pursue microblading is the time saved from meticulously filling in eyebrows with brow pencils, gels, and pomades. Microblading is an innovative, semi-permanent cosmetic tattooing procedure that uses a scalpel-like tool with fine needles in a row to create hair-like strokes while depositing pigment into the superficial skin along the eyebrows. There are two main types of medical tattoo pigments: iron oxide and synthetic. Microblading utilizes synthetic pigment that does not contain heavy metals and whose non-dispersible property makes it difficult to retain in the skin, and hence, qualifies it as semi-permanent in nature. The pigment is deposited into the papillary layer of the dermis and usually fades by 12-18 months [2]. The result is a soft and subtle brow as compared to the opaque and bright brow from traditional tattoos.

While microblading has its cosmetic purposes, it also frequently functions to help patients suffering from alopecia areata, scars, trichotillomania, hypothyroidism resulting in madarosis, burns, chemotherapy-induced hypotrichosis, and a variety of other disorders [2,3]. Microblading is advertised as having very few complications [2]. However, it bears risks similar to other tattoo procedures including local inflammation, infection, allergic contact dermatitis, and chronic granulomatous reactions that patients must be made aware of prior to the procedure [3].

## Case presentation

A 49-year-old Hispanic female with a past medical history of type II diabetes mellitus and hypertension presented with a complaint of a pruritic rash over her eyebrows which occurred a month after she had done microblading to both brows in Mexico 16 months prior. She stated she had two intralesional steroid injections administered over two months by the same doctor in Mexico who performed the initial microblading and had noticed some temporary improvement. She also tried Quadriderm cream, sulfur cream, and mupirocin ointment with no resolution of her symptoms. She mentioned having a red lip liner tattoo in the past with no local reactions or complications.

**Figure 1:**
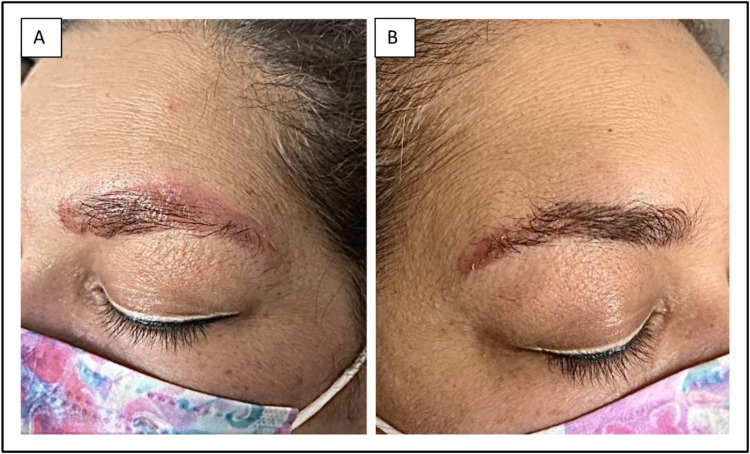
Erythematous and indurated plaques over the entire left eyebrow (A) and over the lateral half of the right eyebrow (B).

On exam, she had erythematous and indurated plaques over the entire left eyebrow and a smaller plaque with crusting over the right lateral eyebrow (Figures 1A, 1B). The remainder of the physical exam was unremarkable. The clinical presentation and patient history suggested a possible diagnosis of chronic contact dermatitis and treatment was initiated with Clobetasol 0.05% topical ointment twice daily.

**Figure 2:**
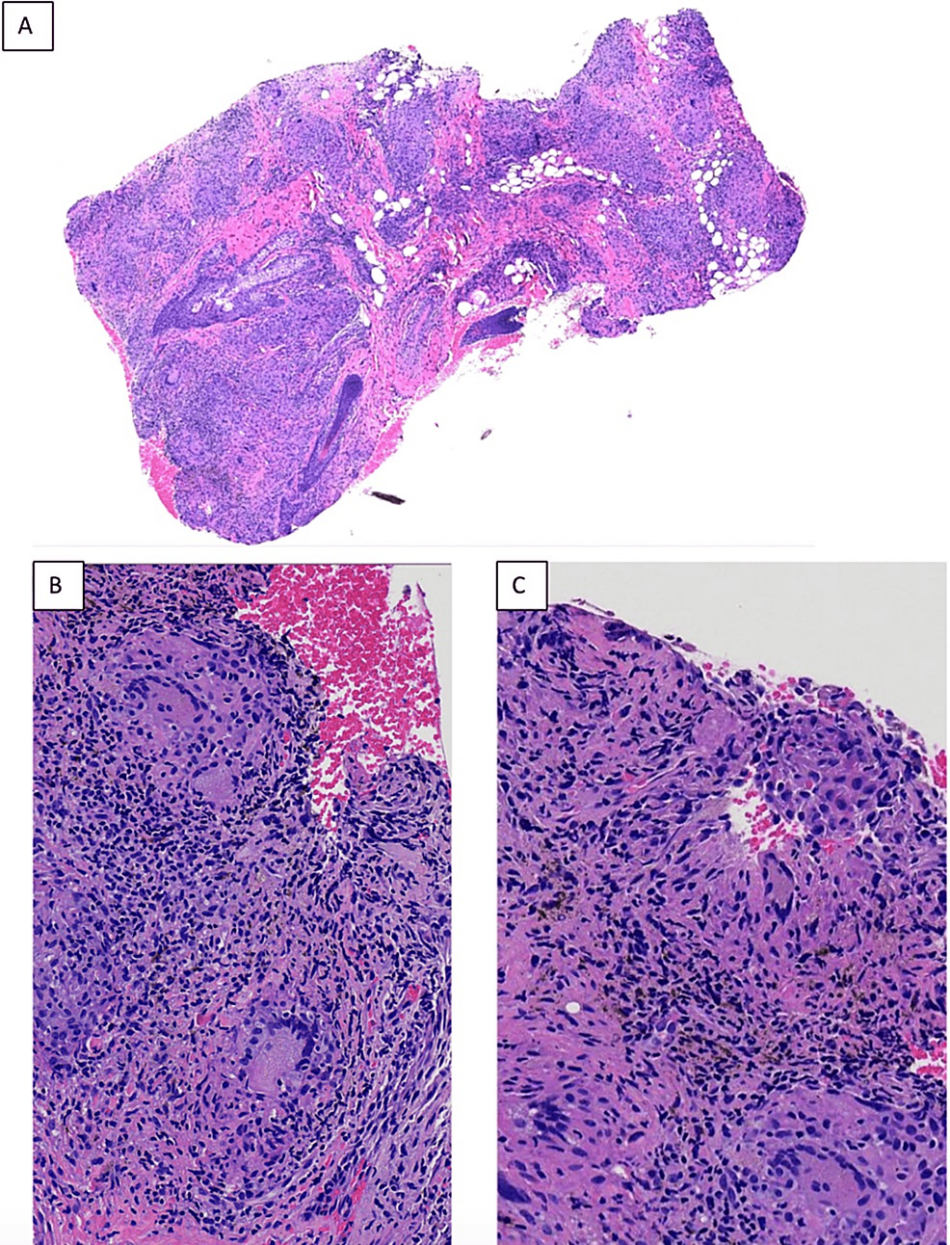
(A) 4x magnification, (B) 10x magnification, and (C) 40x magnification of tissue sample biopsy. Histology shows fine-black intracellular particulate material in the cytoplasm of histiocytes in the papillary dermis, but no polarizable material was evident.

At her two-week follow-up, the patient reported compliance with treatment and experienced some mild improvement of the pruritis and crusting, but the rash was still present. Because of the lack of significant improvement, intralesional Kenalog 0.5mL for a total of 2.5 mg/mL was injected into both eyebrows. Four weeks later, the patient had no noticeable improvement. It was then decided to proceed with a biopsy, and a 2-mm punch biopsy was performed over the left eyebrow. Results of the biopsy showed discrete granulomas in the lower portions of the dermis, most of which were naked with no surrounding cuff of mononuclear cells. There was fine-black intracellular particulate material in the cytoplasm of histiocytes in the papillary dermis, but no polarizable material was evident (Figures 2A-2C). PAS, Fite, and tissue gram stains revealed no microorganisms. Given the histological results, a chest x-ray was done and was unremarkable and the serum level of angiotensin-converting enzyme (ACE) was checked and was within normal range, thus reducing the likelihood of sarcoidosis.

Given the biopsy results, negative chest x-ray findings, and normal ACE levels, the patient was diagnosed with a chronic granulomatous dermatitis secondary to microblading tint. The treatment plan at this time includes a trial of 2mL of 5.0 mg/mL intralesional Kenalog injections every four to six weeks and reassessment of treatment if there is no significant improvement after six rounds.

## Discussion

Microblading is a service being offered across many beauty salons and med spas and is commonly performed by licensed estheticians [4]. It has become a prominent procedure over the last few years. As demand for microblading services rises, there is also a rise in underqualified professionals performing the procedure. The tools required to perform microblading and its variations, including nano-blading, 3D or 6D brows, and micro-shading, may be minimal, but the procedures require technical mastery [2]. Due to its novelty, there is limited research on its side effects and long-term complications. While only a few perform microblading, dermatologists should be aware of this procedure and its serious risks [2].

Although patients are consulted prior to the procedure, undergo a pre-procedure analysis of their skin, and the pigment is only applied to the superficial dermis, there can be unintended consequences [2]. There are only a few cases reported in the medical literature of chronic dermatologic complications occurring secondary to semi-permanent eyebrow tattooing, including cutaneous sarcoid, sarcoid-like reactions, koebernization, and delayed granulomatous reactions documented in the literature [1,3,4]. Leight-Dunn et al. reported spontaneous resolution after six months of a granulomatous reaction that occurred three months after microblading [4]. Klontz et al. attempted treatment with topical corticosteroids, 5% imiquimod cream, and erbium: YAG and 595-nm pulsed-dye laser therapy with limited improvement and continued patient discomfort [5]. Another case of a delayed granulomatous reaction to tattoo ink that presented after 1.5 years was successfully treated with Dermojet and triamcinolone acetonide A10 1:1 diluted with lidocaine [6]. Kluger reports options for treatment in granulomatous tattoo reactions include clobetasol propionate for three months, oral hydroxychloroquine, oral tetracyclines, allopurinol, and oral methotrexate [7]. In another report, five cases of delayed granulomatous reactions to tattoo pigment in the eyebrows were successfully treated with multiple rounds of topical and intralesional corticosteroid injections. Laser removal treatment was not routinely recommended due to systemic anaphylaxis from the release of pigment particles into the bloodstream [4]. Therefore, we chose to start with multiple rounds of minimally invasive intralesional Kenalog injections and will reassess after a maximum of six rounds if no improvement is noted.

## Conclusions

As in our case, it can be very challenging when chronic reactions secondary to microblading tint present to dermatologists. Previous case studies have reported the resolution of granulomatous tattoo reactions within three to six months with intralesional steroid injection. However, our patient has been experiencing a recurrence of symptoms for over 18 months despite prolonged treatment. These cases highlight the potential for severe and chronic reactions that can greatly affect a patient’s life and be financially, physically, and mentally taxing.

Our case emphasizes the need for qualified professionals to perform microblading as these individuals have the knowledge, tools, and training to prevent, recognize, treat, and educate patients on the delayed skin pathologies associated with microblading. Clients considering microblading deserve to be counseled appropriately about the physical and psychological risks associated with undergoing this cosmetic procedure.
